# A Case of Zinc Phosphide-Induced Acute Fulminant Liver Failure

**DOI:** 10.7759/cureus.48485

**Published:** 2023-11-08

**Authors:** Varun Daiya, Nishtha Manuja, Abhinav Kadam, Sourya Acharya, Sunil Kumar

**Affiliations:** 1 Medicine, Jawaharlal Nehru Medical College, Datta Meghe Institute of Medical Sciences (Deemed to Be University), Wardha, IND

**Keywords:** coagulopthy, coma, hepatic failure, poisoning, zinc phosphide

## Abstract

The use of rodenticides such as zinc phosphate is common in tropical countries. However, it has a toxic effect on humans when consumed or absorbed accidentally or deliberately. Although the adverse effects often only last for a short period, acute or fulminant liver failure can occur in few patients. Because the chemicals can create a wide variety of symptoms, it is essential to investigate the progression of symptoms from mild to severe so that treatment protocols can be understood and patients can receive appropriate care. In this report, we detail a case of rodenticide poisoning in a middle-aged man who, initially, had only minor symptoms but ultimately developed fulminant liver failure.

In this example, we discuss the case of a 40-year-old man who intentionally consumed 10 gm of rat poison (zinc phosphide (ZnP)) and reported to our department with a complaint of nausea and three episodes of vomiting. A neurological evaluation showed that the patient had a Glasgow Coma Scale score of 9/15 (Eye(E): 2; Motor(M): 4; Verbal(V): 3). Doll’s eyes were present, and the patient’s pupils were semi-dilated, sluggishly reacting to light. The plantars were bilateral extensor. In the subsequent four hours, the patient developed a deep coma. The patient’s lack of awareness, coagulopathy, and abnormal liver enzyme values all pointed to acute fulminant liver failure. His condition improved with supportive therapy over a period of three weeks.

## Introduction

Zinc phosphide (ZnP) is a highly potent toxin present in commercially available rodenticides and pesticides. Its poisoning is more common in Asia, where the drug is often ingested intentionally as a method of suicide [1]. The compound is readily accessible to subsistence farmers in the tropics because of its low cost [2].

Accidental ingestion of ZnP can result in death via two other routes of entry into the body: inhalation and skin contact [1]. Acute exposure to ZnP is fatal for humans [1,2].

Most ZnP-related deaths have been caused by circulatory collapse, organ toxicity, shock symptoms, acute pulmonary edema, or impaired cellular respiration [3,4].

Acute liver failure (ALF) is characterized by a rapid deterioration of liver function in the absence of preexisting liver disease. Jaundice, decreased mental status, and coagulopathy are all signs of it. ALF caused by drug-induced hepatitis is a leading cause of death and disability among adult individuals. ZnP can cause liver failure and serious damage [5].

This case of ZnP-induced ALF was reported in Acharya Vinoba Bhave Rural Hospital, which is a tertiary care center in Wardha, India.

## Case presentation

**Figure 1:**
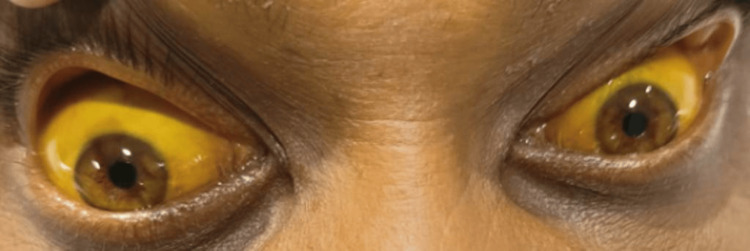
Clinical view of icterus

A 40-year-old man presented to the Department of Internal Medicine, Acharya Vinoba Bhave Rural Hospital, with an alleged history of consuming 10 gm of rat poison (ZnP) six days prior. He had yellowish discoloration of the eyes for the past two days and six episodes of vomiting on the day of admission (see Figure 1).

There was no history of seizures, abdominal pain, bleeding, dyspnea, hemoptysis, or breathlessness. Potassium permanganate gastric lavage was given to the patient. He was admitted and observed. On examination, the patient’s blood pressure was 140/90 mmHg, his pulse was 100 beats per minute, and his respiratory rate (RR) was 24.

Diagnostic assessment

The patient’s routine hemogram, liver function test, coagulation profile, and kidney function tests are discussed in Table 1. On the second day of admission, the patient became drowsy. Repeat examination revealed a pulse rate of 54 beats per minute. His blood pressure was 140/94 mmHg, and icterus was present. An abdomen examination revealed a liver span of 4 cm in the right mid-clavicular line. Arterial blood gas (ABG) analysis revealed no abnormal changes; fundus examination showed signs of early papilledema.

**Table 1:** Laboratory investigations MCV: Mean corpuscular volume; TLC: Total leucocyte count; ALT: Alanine transaminase; APTT: Activated partial thromboplastin time; PT: Prothrombin time; INR: International normalized ratio

Lab Parameters	Observed Value	Normal Range
Hemoglobin	15 gm%	13-17 gm%
MCV	86.3 fL	83-101 fL
TLC	4,100 cells/cu mm	4,000-10,000 cells/cu mm
Platelets	1.30 lakh/ cu mm	1.5-4.1 lakh/ cu mm
Urea	67 mg/dL	19-43 mg/dL
Creatinine	1.7 mg/dL	0.66-1.25 mg/dL
Sodium	142 mmol/L	137-145 mmol/L
Potassium	4.2 mmol/L	3.5-5.1 mmol/L
Alkaline phosphatase	417 U/L	38-126 U/L
ALT	694 U/L	50 U/L
Aspartate aminotransferase	541 U/L	17-59U/L
Albumin	2.4 g/dL	3.5-5 g/dL
APTT	51 sec	29.5 sec
PT	46.3 sec	<20 sec
INR	4.22	1-1.5
Unconjugated bilirubin	3.4 mg/dl	0.0- 1.1 mg/dl
Conjugated bilirubin	15.6 mg/dl	0.0-0.3 mg/dl
Total bilirubin	29 mg/dl	0.2-1.3mg/dl

The diagnosis of ALF was made based on the presence of coma, coagulopathy, and abnormal liver enzymes. A brain CT scan revealed no abnormal changes. Sonographically, the liver’s right, left, and caudate lobes displayed hyperechoic regions that were moderately hyperdense and had irregular shapes around the periphery. The patient also had mild ascites and bilateral pleural effusion (right: 200-300 ml; left: 150-250 ml). The MRI of the brain without contrast revealed no obvious abnormality in the brain parenchyma.

The patient was managed by 25% intravenous dextrose 100 ml thrice daily at a rate of 100 ml/hr, endotracheal intubation and mechanical hyperventilation for increased intracranial pressure, intravenous mannitol 100 ml thrice daily, lactulose 30 ml thrice daily, high-volume bowel cleanses, 15 units of fresh frozen plasma, proton pump inhibitors, and prophylactically intravenous cefotaxime 2 g thrice daily was given. Vitamin K and N-acetylcysteine infusion (NAC) was also given at 150 mg/kg for one hour, 50 mg/kg over the next four hours, and 100 mg/kg over the following 16 hours.

The patient improved over the next four days, and recovery was achieved within a week.

Follow-up and outcomes

The follow-up lab investigations revealed considerable improvement in the assessed parameters (Table 2).

**Table 2:** Follow-up investigations INR: International normalized ratio; PT: Prothrombin time; APTT: Activated partial thromboplastin time; AST: Aspartate transaminase; ALT: Alanine transaminase

Parameters	During Discharge	During Follow-Up
INR	1.02	1.02
PT	18 sec	16 sec
APTT	32 sec	30 sec
Total bilirubin	23 mg/dl	14 mg/dl
Unconjugated bilirubin	2. mg /dl	1 mg/dl
Conjugated bilirubin	20.9 mg /dl	13 u/l
AST	230 u/l	142 u/l
ALT	198 u/l	99 u/l

## Discussion

Literature on ZnP poisoning and its associated symptoms has been published in Asian countries, although reports of its association with acute or chronic liver failure are rare [1,5-8]. Common signs of ZnP poisoning include nausea, vomiting, abdominal pain, shortness of breath, low blood pressure, rapid heart rate, abnormal heart rhythms, agitation, hallucinations, depression, and coma [9,10]. The most common laboratory findings include altered hemogram, electrolyte imbalance, and abnormal serum enzymes [10,11].

Our patient reported nausea and vomiting, but other relevant symptoms were absent. The patient’s age, higher serum bilirubin value, presence of encephalopathy, deranged INR , and associated drug toxicity all pointed to ALF according King's College Criteria [12].

Gokdemir et al. had observed that the death rate for those who develop increased liver enzymes following ZnP poisoning may double [13]. Saleki et al. had studied postmortem liver biopsies from people who had overdosed on ZnP and found evidence of liver injury in all of them involving congestion to necrosis [14].

Inhibition of oxygen uptake in liver mitochondria, inhibition of the adenosine diphosphate uncoupling site, ion-stimulated respiration, and effects on pyruvate malate, succinate, glycerophosphate, and ascorbate cytochrome biomolecules have all been proposed as potential mechanisms of phosphide poisoning. Within five hours of exposure, mitochondrial membrane undergoes significant morphological changes including inhibition of oxidative respiration, inhibition of cytochrome C oxidase system, and interaction with hydrogen peroxide to produce the highly reactive hydroxyl radical, all of which contributes to oxidative damage and cell death [15].

In the view of hepatic encephalopathy, non-aggressive bowel cleansing and/or ammonia-lowering treatment has been advised [16]. Our patient underwent high volume bowel cleansing for the same. The most immediate treatment for coagulopathy induced by fulminant hepatic failure is intravenous vitamin K infusion, followed by other supportive treatments such as fresh frozen plasma. An NAC infusion was given to our patient as it has anti-inflammatory, inotropic, and vasodilatory properties. Its beneficial effect could be explained by enhancing the microcirculation’s oxygen supply, which is disrupted by liver failure [17]. NAC recipients exhibited lower mortality, lower peak aspartate transaminase (AST) and alanine transaminase (ALT) values, and shorter hospital stays in a recent study on 100 patients with liver failure brought on by ZnP poisoning [18].

Liver transplantation may be helpful for patients who do not improve after receiving supportive treatments [19,20]. Our patient was treated symptomatically and exhibited improvement as a result. Given the improvement in our patients’ situation, liver transplantation was put on hold.

## Conclusions

To conclude, acute fulminant hepatic failure can result from ZnP overdose. We present a rare but archetypal instance of severe fulminant hepatic failure caused by ZnP poisoning in a male patient who recovered after careful critical care therapy. Treating doctors should actively monitor such patients to identify a downhill trend as early as possible to prevent potential fatal implications in otherwise benign-looking cases of ZnP poisoning.
